# Variation in the Male Pheromones and Mating Success of Wild Caught *Drosophila melanogaster*

**DOI:** 10.1371/journal.pone.0023645

**Published:** 2011-08-17

**Authors:** David Scott, Alicia Shields, Michaela Straker, Heidi Dalrymple, Priya K. Dhillon, Singh Harbinder

**Affiliations:** 1 Department of Biological and Physical Sciences, South Carolina State University, Orangeburg, South Carolina, United States of America; 2 Beckman Center, Stanford University School of Medicine, Stanford University, Stanford, California, United States of America; 3 University Centre, School of Medicine, St. Georges University, Grenada, West Indies; 4 Department of Biology, Delaware State University, Dover, Delaware, United States of America; University of Texas Arlington, United States of America

## Abstract

*Drosophila melanogaster* males express two primary cuticular hydrocarbons (male-predominant hydrocarbons). These act as sex pheromones by influencing female receptivity to mating. The relative quantities of these hydrocarbons vary widely among natural populations and can contribute to variation in mating success. We tested four isofemale lines collected from a wild population to assess the effect of intrapopulation variation in male-predominant hydrocarbons on mating success. The receptivity of laboratory females to males of the four wild-caught lines varied significantly, but not consistently in the direction predicted by variation in male-predominant hydrocarbons. Receptivity of the wild-caught females to laboratory males also varied significantly, but females from lines with male-predominant hydrocarbon profiles closer to a more cosmopolitan one did not show a correspondingly strong mating bias toward a cosmopolitan male. Among wild-caught lines, the male-specific ejaculatory bulb lipid, *cis*-vaccenyl acetate, varied more than two-fold, but was not associated with variation in male mating success. We observed a strong inverse relationship between the receptivity of wild-caught females and the mating success of males from their own lines, when tested with laboratory flies of the opposite sex.

## Introduction

Hydrocarbons on the cuticle of *Drosophila melanogaster* are sexually dimorphic and those on both males and females act as pheromones [1], [2]. Female cuticular hydrocarbons are predominantly 27 or 29 carbon dienes and stimulate male courtship [1]. The predominant hydrocarbons on males are 23 or 25 carbon monoenes that act as pheromonal signals to help induce female receptivity to mating [1], [2]. Extensive natural intraspecific variation in cuticular hydrocarbons on male *D. melanogaster* has been documented [1], [3]. Speciation may be primarily driven by intrademic female choice and sexual selection of courtship cues in several *Drosophila* species [4]. Recent evidence indicates that in the cactophilic desert species *D. mojavensis* mating preferences are associated with variation in cuticular hydrocarbons and that sexual selection based on cuticular hydrocarbon variation may drive divergence between populations [5], [6], [7]. However, there has been little study of female preferences and potential sexual selection based on cuticular hydrocarbon variation in *D. melanogaster* (see [2]), especially variation within local populations.

The reported variation in cuticular hydrocarbons on *D. melanogaster* is primarily due to differences in the quantities of the two male-predominant hydrocarbons, (*Z*)-7-tricosene ((*Z*)-7-C_23:1_) and (*Z*)-7-pentacosene ((*Z*)-7-C_25:1_) and associated C23 and C25 monenes that are present in very small quantities [1], [8]. For the common laboratory strain Canton-S and other North American strains the primary male-predominant hydrocarbon is (*Z*)-7-C_23:1_, which makes up about 50% of the total cuticular hydrocarbons [1]. This is considered a cosmopolitan hydrocarbon profile. For the Tai-Y strain (a laboratory strain originally collected on the Ivory Coast of Africa, see [1]) (*Z*)-7-C_23:1_ and other C23 hydrocarbons are almost absent from both sexes. Instead, (*Z*)-7-C_25:1_ is the male-predominant hydrocarbon [1]. The quantitative difference between Canton-S and Tai-Y males is 6-fold or more for each male-predominant hydrocarbon. Tai-Y profiles are less common than Canton-S and are limited to some African and Caribbean strains [9], [10], but some North American strains, such as Florida-9, have male-predominant hydrocarbon mixes intermediate between Canton-S and Tai-Y [2]. Genes controlling the differences in male-predominant hydrocarbons between Canton-S and Tai-Y are distributed on all three major chromosomes [2], [10], [11].

The Tai-Y and Canton-S strains exhibit mating asymmetries, in that Canton-S females mate faster and almost twice as frequently with Canton-S males as with Tai-Y in no-choice trials, while Tai-Y females mate equally with males of the two strains [2]. The asymmetrical mating of Canton-S females extends to the Florida-9 strain and is not due to differences in courtship intensity - males of all three strains court virgin Canton-S females equally actively [2], [12].

In *D. mojavensis* cuticular hydrocarbons that differ the most among strains from different geographical regions are not necessarily the ones involved in mating preferences [6]. However, the difference in male-predominant hydrocarbons between Tai-Y and Canton-S has been shown to contribute substantially to the mating asymmetry - topical application to Tai-Y of Canton-S male extracts or synthetic (*Z*)-7-C_23:1_ significantly increases the mating speed of Tai-Y males with Canton-S females [2]. The mating advantage of Canton-S over Tai-Y and Florida-9 males with Canton-S females persists when courtship song, a dominant factor in male mating ability [13], [14], has been silenced by surgically removing the wings [2], so the mating difference is not due to variation in courtship song components that would remain undetected by measurement of courtship intensity. Finally, Canton-S and Tai-Y males are able to mate equally often with Canton-S females when both males are placed with a Canton-S female in a small chamber where pheromonal signals from the two males could intermix [2]. Together these results indicate a strong role for male-predominant hydrocarbons in the mating asymmetry, and a limited role, if any, for other courtship signals.

Canton-S and Tai-Y also differ with respect to cuticular hydrocarbons on females that stimulate courtship [1]. However this is unlikely to play a role in the mating asymmetry because Canton-S and Tai-Y males court virgin females from either line as actively, and Canton-S and Tai-Y males mate as successfully with Tai-Y females [1], [14]. More generally, greater courtship intensity does not necessarily lead to greater mating success - even when female hydrocarbon profiles are manipulated sufficiently to produce variation in courtship intensity, this does not necessarily produce variation in mating success [15], [16], [17].

Less extreme variation in male-predominant hydrocarbons than that between Canton-S, Tai-Y and Florida-9 may also influence mating. Females from the laboratory strain N2 (which is a wild type revertant of the *desat* mutation and similar to Canton-S) mate faster when males are perfumed with less than 100 ng of additional (*Z*)-7-C_23:1_ [18]. They also respond to small quantitative differences in (*Z*)-7-C_23:1_ that surgically antenna-less females are unable to detect [18]. These data suggest that relatively minor natural variation in male-predominant hydrocarbons could bias female mating receptivity. Two other monoenes, (*Z*) 5-tricosene ((*Z*)-5-C_23:1_) and (*Z*) 9-pentacosene ((*Z*)-9-C_25:1_) could also influence female receptivity because they have pheromonal effects on courtship between normal males and mosaic males[19] although topical application of (*Z*)-5-C_23:1_) to females does not alter their attractiveness to males [15].

In addition to male-predominant hydrocarbons, a male-specific ejaculatory duct lipid, (*Z*) - octadecenyl acetate (*cis* vaccenyl acetate (cVA)) [20], [21] may also have pheromonal properties that affect mating behavior. Males transfer cVA to females during mating, along with the seminal fluid [22]. Females subsequently expel most of the cVA onto the substrate, where it may interact with food as an aggregation pheromone [23]. However, some cVA remains in females for a few hours after mating [22], [24], [25]. When topically applied to virgin females, cVA acts as a courtship inhibitor [26] and can induce aversive learning when associated with components of a female's hydrocarbon profile [27], but it does not seem likely that cVA acts as an antiaphrodisiac during courtship [1], [28].

In tests with females, stimulation of the specific olfactory receptor Or67 by cVA promotes receptivity to males [29] so the presence of cVA during courtship would likely enhance female receptivity. Almost all cVA from males is found in the ejaculatory bulb [20], [21], [28]. However, direct sampling of live males' cuticles produces a qualitative signal from cVA in the anal-genital region, but not the thorax [30]. The cVA in the anal-genital region of the male's cuticle is especially localized on the tip of the fly's penis [25]. Everaerts et al. [31] detected no cVA on the cuticle of naïve virgin males, but it was present on the cuticle of males separated from females immediately after mating began. Transfer of cVA to females in the ejaculate does not begin until 1-3 minutes after mating begins [28], so cVA present on the male at the initiation of mating would seem to have been accumulated during courtship.

Here, we use wild-caught flies to evaluate the effects of natural variation of male-predominant hydrocarbons and cVA on mating success. We assess (1) whether the mating discrimination by Canton-S females observed in prior experiments [2] extends to flies from a variable natural population with less divergent male-predominant hydrocarbon mixes and (2) whether females from a natural population with a variable hydrocarbon mix show a Canton-S like distinction between Canton-S and Tai-Y. We also examine whether variation in mating effectiveness among wild-caught males is associated with variation in superficial cVA.

## Materials and Methods

### *Drosophila* Lines

Test flies were from the Canton-S and Tai-Y laboratory strains. Both are wildtype and indistinguishable with respect to visible morphology. The Canton-S strain is a common laboratory stock obtained from the Indiana University Stock Center, Bloomington, IN 47405. The Tai-Y strain is from an isofemale line initially collected from the Ivory Coast, Africa [1]. Stocks of both strains have been maintained under the same laboratory conditions (see below) for over 20 years. Canton-S and Tai-Y males differ dramatically in the amount of (*Z*)-7-C_23:1_ and (*Z*)-7-C_25:1_ on their cuticles, but little, if any, in the amounts of other cuticular hydrocarbons [1], [11]. Florida-9 (obtained from the Drosophila Stock Center, Bloomington IN), is a laboratory stock initially collected in Florida and is intermediate between Canton-S and Tai-Y for male-predominant hydrocarbons [2].

Wild *D. melanogaster* were collected from banana baits near the biology building on the South Carolina State University campus in Orangeburg SC. Three banana baits were placed approximately 50 m from each other. Ten isofemale lines, designated SC1 through SC10, were started from these flies. Each line was tested to ensure that it was *D. melanogaster* by crossing wild-caught males with laboratory *D. melanogaster* females (to determine whether the F_1_ was fertile), and by gas chromatographic analysis (see below) of male and female hydrocarbon profiles. The isofemale lines have been cultured in isolation in half-pint bottles or 40 ml vials without intermixing since they were initially collected. In addition to the initial chromatographic analysis, the hydrocarbon profiles were analyzed again about two years later when the flies were used for mating experiments.

### Fly Culture and Mating Assays

Flies were reared at 23°C and 12:12 L:D cycle on cornmeal-molasses-agar medium sprinkled with live yeast, under uncrowded conditions. All flies used in mating experiments were raised in 40 ml vials. They were collected and separated by sex within 16 hr of eclosion under light ether anesthesia. Reared at 23°C, virtually all females less than 16 hr old are virgins (nonvirgins were not used – see below). After collection, flies were aged for 4–5 days before testing. Females were maintained individually, males in groups of two or occasionally three males each (about 2% of males were stored in groups of 3), in 10 ml test tubes containing 2 ml food medium sprinkled with live yeast.

Storing males in groups of five has been shown to reduce subsequent courtship in at least one strain [32]. Svetec et al. [32] did not observe any effect on courtship for Canton-S males stored in groups, and their males were stored in groups of five and ours usually in groups of two. Nonetheless, we tested our laboratory males in homogametic matings to determine whether storing them in groups of two would reduce mating success. The results are shown in Table 1. Storing males in groups did not reduce mating success for either of the two laboratory lines. Canton-S was not affected by storage in groups, and Tai-Y mated more successfully after being stored in groups, resulting in a significant interaction between strain and storage method. When mating success was measured by our mating index, which combines time to initiate mating and percentage mating (see below), Canton-S and Tai-Y were not significantly different when stored in groups of two.

**Table 1:** Mating Speed and Mating Index (± SEM) for homogametic pairings using Canton-S and Tai-Y males stored singly or in groups of two.

Mating Measure	Strain	Single Storage	Group Storage
Mating Speed	Canton-S	11.3±1.14, *N* = 46	11.4±0.67, *N* = 79
	Tai-Y	13.5±1.24, *N* = 39	9.5±0.67, *N* = 78
Mating Index	Canton-S	13.8±1.41, *N* = 46	13.2±0.77, *N* = 79
	Tai-Y	18.2±1.67, *N* = 39	12.8±0.97, *N* = 78

ANOVA Mating Speed: No significant main effects for Strain or Storage, for the interaction *F_1,238_* = 8.14, *P* = 0.005.

ANOVA Mating Index: No significant main effects for Strain or Storage, for the interaction *F_1,238_* = 6.88, *P* = 0.009.

Mating assays were conducted within 3 hr of lights-on at room temperature (23°–25°C) with 4–5 day old flies. Female storage tubes were examined for larvae at the time they were tested. Only virgin females were used in the mating experiments. Flies to be tested were aspirated without anesthesia into 0.3 cm^3^ mating chambers. These chambers were made with flat-bottomed holes drilled into a circular Plexiglas wheel covered by a Plexiglas plate, 20 chambers per wheel [2]. Single females were first placed in all chambers to be used, a maximum of 20, then a male was added to each chamber. Timing was started for each chamber when the male was added. The test was continued for 30 minutes, during which the individual pairs of flies in each chamber were monitored continuously by two observers. Mating speed for each pair was measured as the time it took for copulation to begin. In tests that were continued for 45 minutes, only an additional 2% of flies mated, so we considered 30 minutes sufficient for effective courters to mate. The percentage of pairs mating during a trial was also recorded. Of the laboratory strains, only Canton-S females were used in mating tests with wild-caught males, because they discriminate between Canton-S and Tai-Y males on the basis of hydrocarbon profiles but Tai-Y females do not [2].

### Mating Index

In mating experiments such as the ones we conducted for this report, either the percent mating or the mean time required to initiate mating ( =  mating speed) have been reported, or both have been included as separate measures (see [2], [18]). However, in some cases neither of the two measures gives a clear representation of mating success, for either of the two following reasons. First, the two measures may contradict each other, for example if a higher percent mated in a group with slower mating speed. In this case any decision about which of the two parameters is the most meaningful would be subjective. Second, even if the two measures may be non-contradictory, neither difference may be statistically significant on its own, so real mating differences may be obscured.

To minimize these limitations, we formulated a mating index (MI) that combines both measures. Our MI increases the time to mating for each pair (*t*) by a constant obtained from the proportion of pairs that mated (*p*) for that specific pairing combination, such that the higher the percentage mating, the smaller the correction factor. The MI for each individual pair was calculated by: *t* (2 – *p*). There is no variance in *p*, so the MI for each pairing combination has the same distribution characteristics as the time to mating. If all pairs were to mate, the MI would equal the time to mating because *p* would equal 1. If only a proportion of pairs mate (as in most practical cases), each mating pair's time to mating is increased by an amount that is inversely proportional to the percent mating for that pairing combination. Both the mating speed and MI are inversely proportional to overall mating success so that the higher the mating success, the smaller the mating speed and MI. We have included mating speed and percent mating data for comparison. However, combining them into an MI provides a sensitive measure of overall mating success that sums the two components and thus increases resolution.

### Analysis of Cuticular Hydrocarbons

For quantitative analysis, hydrocarbons were removed from individual 4–5 d old males by washing the males for 1 minute in 100 ul of hexane. Just prior to the wash, 100 ng of n-eicosane (C_20_) was added to the hexane as an internal standard. A 1 minute wash removes a maximal amount of cuticular hydrocarbons and a limited amount of cVA [33]. A short hexane wash effectively removes superficial cVA (on or near the surface) that might be available for release even if it lacks the resolving power of more sophisticated techniques that directly sample the surface of the fly's cuticle [24], [25], [30], [31]. The ejaculatory bulb of adult virgin Canton-S males contains about 1500 ng of cVA, but 5 second and 1 minute washes remove only about 8 ng and 50 ng, respectively [20], so ejaculatory bulb cVA is not readily removed by a short wash. The amount of cVA on the cuticle of a naïve male seems to be relatively small, [24], [25], [28], [30], [31], but some may be near the surface of the fly (for example in the reproductive tract near the tip of the penis [25], [30]) and potentially available for release as a pheromone during courtship. In our own analyses, a 30 second hexane wash removed 63±28 ng, *N* = 6 of cVA from Canton-S males in one experiment; in another 124±25 ng, *N* = 6. There is clearly a substantial amount of variation both within and between samples, but when these two data sets are pooled, the amount of cVA removed by a 30 second wash is 93±20 ng, *N* = 12, comparable to the amounts removed by a 1 minute wash shown in Table 2). At least some of the cVA in a wash is likely to be leached from the ejaculatory bulb or reproductive tract [20], [23] but the relatively small amount of cVA in a wash and the similarity of 30 second and 1 minute washes indicates the presence of superficial cVA. The cVA present in a wash would include any of the compound on or near the external cuticle.

**Table 2:** Mean ng (± SEM) of male-predominant hydrocarbons, associated 23 and 25 carbon monenes and CVA from laboratory and wild-caught males.

Line (*N*)	(*Z*)-7-C_23:1_	(*Z*)-5-C_23:1_	(*Z*)-7-C_25:1_	(*Z*)-9-C_25:1_	cVA
Laboratory Lines (not included in ANOVA)					
Canton-S (31)	454±25	62±15	88±9	34 + 12	90±12
Tai-Y (30)	29±3	50±6	720±25	59 + 8	88±12
Florida-9 (15)	146±6	25±3	467±17	93±10	103±20
Wild-caught Lines					
SC3 (18)	531±34 *a*	67±5	220±25 *a*	70±7 *nsd*	166±31 *nsd*
SC5 (17)	380±16 *b*	60±8	138±10 *b*	48±8 *a*	104±21 *a*
SC7 (19)	369±26 *b*	48±4	229±28 *a*	64±13 *nsd*	235±25 *b*
SC9 (17)	290±26 *c*	46±7	320±37 a^*^	90±15 *b*	91±23 *a*
ANOVA: *F*_3,67_	14.30,*P*<0.001	2.70,*P* = 0.053	8.13,*P*<0.001	2.88,*P* = 0.042	4.91,*P* = 0.004

SC line means that differed from each other by one-way ANOVA with Tukey *post hoc* tests at the 0.05 level are designated by different letters. Those marked by an asterisk formed a third distinct group (*P*<0.05) by Fisher's LSD *post hoc* test. Those marked *nsd* did not differ significantly from any of the others. Canton-S, Tai-Y and Florida-9 were included for comparison and were not included in the statistical analysis.

We have included cVA data to illustrate variation in the amount of cVA near the surface of the fly, in any superficial anatomic location, and potentially available for release during courtship. This is not meant to imply that it was present on the cuticle, or that it would have been released had the flies been courting. It simply implies that it was near the exterior of the fly. Care was taken during the wash not to bump the fly carcass with the pipette used to remove hexane, as this could force internal lipids into the wash.

Samples were stored at −20°C until they were analyzed. Before analysis, individual washes were evaporated to about 1 microliter under nitrogen. The entire sample was then injected into a Varian 3300 gas chromatograph equipped with a flame ionization detector and a Restek RT-1 30 m X 0.32 mm ID fused silica column. Peak areas were quantified with a Varian 4400 integrator.

To determine that wild-caught flies were *D. melanogaster*, hydrocarbons were removed in one wash from 10–20 adult males or females and treated as above (*D. melanogaster* has a characteristic hydrocarbon profile and is strongly sexually dimorphic for cuticular hydrocarbons, whereas the nearly identical sibling species *D. simulans* is not [1]). These bulk washes were also used for initial assessment as to whether a line differed substantially from Canton-S. Other details of gas chromatographic procedure are described in Scott and Richmond [11].

### Statistical Analysis

ANOVA was done with SYSTAT 12 (Systat Software, Richmond, CA). All data were transformed to normalize the distributions. Mating data were log transformed, hydrocarbon and cVA data were square root transformed. Normality of the distributions was checked by Anderson-Darling tests. Normality was obtained in all hydrocarbon and cVA data. For mating data, normality was achieved in 15 out of 18 pairing combinations and improved in the remaining three. Along with ANOVA, normality was tested by Kolmogorov-Smirnov tests and homogeneity of variances was tested by Levene's test. Tukey adjustments were used in *post hoc* tests where variances were equal. In one ANOVA variances were unequal and a Games-Howell *post hoc* test was used. Contingency *X* ^2^ for percentages were calculated according to Box et al. [34]. For mating data, we analyzed matings that occurred within 15 minutes as well as for all that occurred within 30 minutes. Both analyses produced the same pattern, although the differences were greater and therefore more easily resolved statistically for 30 minute periods. Only the 30 minute results are included.

## Results

### Male-Predominant Hydrocarbons and cVA

Gas chromatographic analysis of all initial SC isofemale lines showed that male-predominant hydrocarbon profiles varied considerably. Males from seven out of ten wild-caught lines had a relatively typical Canton-S mix, high (*Z*)*-*7-C C_23:1_ (about 400 ng) and low (*Z*)-7-C_25:1_ (about 100 ng) (data not shown, but see SC5, Table 2 for representative quantities). Three lines differed substantially from Canton-S in having either less (*Z*)-7-C_23:1_, more (*Z*)-7-C_25:1_ or both. These three lines along with one of the Canton-S-like lines (SC5) were retained for mating tests – their relatively large differences from Canton-S made them the most likely to exhibit differences in mating effectiveness due to cuticular hydrocarbons. The quantities of male-predominant hydrocarbons and cVA for these lines are shown in Table 2. The statistical analysis was designed to identify differences among wild-caught lines. Canton-S, Tai-Y and Florida-9 data are included for comparison, to illustrate typical hydrocarbon patterns for these lines and to show where the wild-caught flies fall in the range of variation present in the laboratory strains. Canton-S and Tai-Y data were collected at various times prior to the SC line data and Florida-9 data are from Scott [2]. We consider the data from laboratory lines to be comparable to our more recent data from wild-caught lines because periodic checks of hydrocarbon profiles in our laboratory have produced chromatograms that were indistinguishable in side by side comparisons, indicating no substantial variation in hydrocarbon profiles over time (D.S., unpublished results). Also, the reported profiles of Canton-S and Tai-Y from different times and different laboratories have been generally very consistent – Canton-S and Tai-Y display very stable profiles overall [1], [2], [11].

The amounts of (*Z*)-7-C_23:1_ among the SC lines varied almost 2 fold, but all were more like Canton-S than Tai-Y or Florida-9 (Table 2). The amount of (*Z*)-7-C_25:1_ ranged from about normal for Canton-S (SC5) to about 3 times the normal Canton-S amount (SC9). Overall, SC5 was closest to a Canton-S profile, with relatively high (*Z*)-7-C_23:1_ and the lowest (*Z*)-7-C_25:1_. SC9 differed most from Canton-S with the lowest (*Z*)-7-C_23:1_ and the highest (*Z*)-7-C_25:1_ of all four SC lines. The amount of the minor C23 hydrocarbon (*Z*)-5-C_23:1_ did not vary significantly, but did tend to be higher in lines with more (*Z*)-7-C_23:1_. The minor C25 hydrocarbon (*Z*)-9-C_25:1_ varied significantly, but only the highest and lowest amounts were different from each other by *post hoc* tests. As with the C_23_ monenes, (*Z*)-7-C_25:1_ and (*Z*)-9-C_25:1_ followed the same rank order from highest to lowest. The amount of cVA varied significantly with SC7 having a higher quantity than either SC5 or SC9, and SC3 intermediate and not different from any of the others.

### Mating Success Among Homogametic Pairings

Neither mating speed nor percent mating varied significantly for homogametic matings among SC lines (Table 3), and both were similar to homogametic matings with Tai-Y and Canton-S. The MIs also did not vary significantly among SC lines. During all trials, and those below, males courted actively and displayed high frequencies of all the normal components of courtship including orientation, wing vibration, tapping, licking and attempted copulation.

**Table 3:** Mating index, mating speed, and percent mating for homogametic pairings involving Canton-S (CS), Tai-Y (TY) and SC line flies.

Lines (*N*)	Mating Index	Mating Speed	Percent Mating
CS (155)	15.4±0.77	11.3±0.53	64
TY (114)	14.0±0.81	9.9±0.54	68
SC3 (131)	13.4±0.77	9.9±0.57	66
SC5 (127)	13.1±0.63	10.2±0.49	72
SC7 (124)	12.1±0.65	9.1±0.49	68
SC9 (78)	14.5±1.06	10.4±0.76	62
ANOVA: *F*_3,456_	0.89, *P* = 0.45	0.96, *P* = 0.41	*X* ^2^_3_ = 3.64, *P* = 0.30

Mating index and mating speed are presented as mean ± SE. Mating speeds are given in min. *N*  =  number of pairs mated within 30 minutes. SC lines were analyzed by one-way ANOVA with Tukey *post hoc* tests. CS and TY pairings are shown for comparison and were not included in the ANOVA.

### Mating Success of SC Line Males with Canton-S Females in Relation to Male-Predominant Hydrocarbons and cVA

The MI for SC line males with Canton-S females varied significantly (Table 4), but mating speed did not, and percent mating only varied significantly because one pairing combination (with SC7 males) was lower than the other three. Mating speed and percent mating followed nearly the same rank order and combined to produce significant differences among MIs.

**Table 4:** Mating index, mating speed, and percent mating for pairings involving SC line males and Canton-S females.

Lines (♂, (*N*))	Mating Index	Mating Speed	Percent Mating
SC3 (85)	17.1±0.97 *nsd*	11.4±0.65	51
SC5 (80)	13.9±0.87 *a*	9.6±0.60	56
SC7 (58)	19.2±1.53 *b*	11.6±0.93	35
SC9 (95)	14.4±0.84 *a*	10.0±0.59	56
ANOVA: *F*_3,314_	4.70, *P* = 0.003	1.96, *P* = 0.12	*X* ^2^_3_ = 17.4, *P*<0.001

Mating index and mating speed are presented as mean ± SE. Mating speeds are given in min. *N*  =  number of pairs mated within 30 min. Within columns, means followed by different letters differ from each other at the 0.05 level by one-way ANOVA with Tukey *post hoc* tests. Those that were not different from any of the others are designated by *nsd*.

The variation in MIs did not consistently follow the similarity of hydrocarbon mixes to Canton-S. Pairings with SC5 males, whose hydrocarbon mix was nearest to that of Canton-S, had the lowest MI ( =  greatest mating success). However, the SC9 MI was nearly identical to that of SC5 (Table 4, *P* = 1.00, Tukey *post hoc* test) even though the SC9 hydrocarbon mix was the most divergent from Canton-S, especially for (*Z*)-7-C_25:1_ (Table 2). SC7 had a pheromone mix intermediate between SC5 and SC9 and an MI significantly higher than either (SC5, *P* = 0.015 and SC9 *P* = 0.023). SC7 males had the highest amount of superficial cVA, and the lowest overall mating success. SC5 and SC9 had the lowest, and statistically equal, amounts of superficial cVA, and the greatest overall mating success. Overall, even though the hydrocarbon profile among SC line males varied strongly, mating success with Canton-S females did not, except that SC7 had a higher MI and was thus less successful.

### Mating Success of Canton-S and Tai-Y Males with SC Line Females in Relation to Male-Predominant Hydrocarbons and cVA

SC line females tended to have higher MIs with either Canton-S or Tai-Y males than with males from their own strain (Compare Tables 3 and 5), regardless their relative mating propensity with Tai-Y or Canton-S. For pairings with Tai-Y, SC lines all had significantly higher MIs than with SC line males (*P*<0.001 for each by ANOVA with Tukey *post hoc* tests). For pairings with Canton-S males, all females but SC7 had MIs that were significantly higher than with males from their own strain (*P* = 0.013, <0.0001, 0.045 for SC3, SC5 and SC9 respectively). Tai-Y males had lower overall mating success than Canton-S with SC line females, (see male effects, two-way ANOVA, Table 6) and did not mate as successfully with SC line females as with Tai-Y females (Table 3). The highly significant interaction between male and female effects (Table 6) indicates that SC line females did not show a uniform mating bias toward Canton-S males. Overall, the variation in mating success was less for Tai-Y males than for Canton-S, and the only significant pairwise difference among Tai-Y male/SC female matings was that the SC5 MI was lower than SC7 or SC9 (*P*<0.05 for each).

**Table 5:** Mating index, mating speed, and percent mating for pairings involving Canton-S or Tai-Y males with SC line females.

Lines (♀, (*N*))	Mating Index	Mating Speed	Percent Mating
**Canton-S male**			
SC3 (135)	16.2±0.82 *a*	11.5±0.58 *a*	59
SC5 (133)	20.0±0.83 *b*	14.0±0.58 *b*	58
SC7 (142)	11.7±0.74 *c*	9.3±0.59 *c*	75
SC9 (111)	17.7±0.99 *a,b*	12.1±0.67 *a,b*	53
			*X* ^2^_3_ = 22.3, *P*<0.001
**Tai-Y male**			
SC3 (41)	20.0±1.60 *nsd*	11.7±0.93 *a*	29
SC5 (82)	17.4±0.92 *a*	13.0±0.69 *a*	66
SC7 (37)	23.7±1.76 *b*	14.1±1.04 *a*	31
SC9 (55)	23.9±1.57 *b*	14.3±0.94 *a*	33
			*X* ^2^_3_ = 48.8, *P*<0.001

Mating index and mating speed (in min) are presented as mean ± SE. Mating index and mating speed were analyzed by two-way ANOVA with Games-Howell *post hoc* tests. Only within-male comparisons (Tai-Y or Canton-S) are shown. Means that differed at the 0.05 significance level were designated with different letters. Means that did not differ from any of the others were designated with *nsd*. The two-way table is shown in Table 6. Mating percentages were analyzed with a contingency chi-square test.

**Table 6:** Two-way ANOVA of mating indices and mating speeds shown in Table 5.

Source	df	Mating Index		Mating Speed	
		*F-ratio*	*P*	*F-ratio*	*P*
Male line (CS or TY)	1	40.70	<0.001	12.62	<0.001
SC female line	3	3.31	0.020	4.10	0.007
Interaction	3	17.34	<0.001	7.34	<0.001
Error	728				

The MIs for both Canton-S and Tai-Y males varied significantly among the SC line females (Table 5). For Canton-S, mating speed also varied significantly. Among pairings with Canton-S males, the SC5 female/Canton-S male combination had the highest MI and thus the lowest overall mating success, even though SC5 males have a male-predominant hydrocarbon mix most similar to Canton-S. The MI for SC3 and SC5 females differed significantly (*P*<0.01), but males from those two strains have the most similar male-predominant hydrocarbon mix, especially if the ratio of the two hydrocarbons is considered ((*Z*)-7-C_23:1_/(*Z*)-7-C_25:1_ ratio  =  3.0±0.40 for SC3, 2.9±0.16 for SC5). Females from the SC7 line, with male (*Z*)-7-C_25:1_ quantity significantly higher than Canton-S (almost 3-fold greater), had the lowest MI with Canton-S males. The MIs for SC9 females did not differ significantly from either SC3 or SC5, even though the males from SC9 have hydrocarbon mixes that are the most different from Canton-S and significantly different from either SC3 or SC5. Among pairings with Tai-Y males, the highest mating success was with SC5 females, a line with male-predominant hydrocarbons most like Canton-S. Superficial cVA for Canton-S and Tai-Y was not significantly different (Table 2), so did not contribute to the difference in mating effectiveness for males from the two lines.

### MIs of Canton-S Males by SC Line Females and SC Line Males by Canton-S Females

Our results show an inverse relationship between the MIs of the two reciprocal Canton-S/SC line pairings (Tables 4, 5). For example, the SC5 female/Canton-S male MI is the highest of the four pairing combinations with SC line females, while the Canton-S female/SC5 male MI is the lowest of the combinations with SC line males. When the MIs of the other three pairings with SC line females are arranged in descending order, they correspond to an ascending order of MIs for pairings with SC line males. A two-way ANOVA of data compiled from SC line male/Canton-S female (Table 4) and Canton-S male/SC line female (Table 5) confirmed this inverse relationship, showing a highly significant interaction between Canton-S and SC line (Table 7). The same pattern holds in a comparison that includes only mating speed (*F*_3,831_ = 10.47, *P*<0.001 for the interaction), so the effect is robust. It also holds for mating percentages, but variation in the percentages is almost entirely due to one pairing combination in both of the data sets. This strong inverse relationship is unrelated to how closely the hydrocarbon profile of the SC line males resembles Canton-S males, or to the quantity of superficial cVA.

**Table 7:** Two-way ANOVA testing interactions with Canton-S male/SC female and SC male/Canton-S female pairings shown in Table 4 and Table 5, Panel 1.

Source	*df*	*F*-ratio	*P*
Canton-S effects	1	0.19	0.663
SC line effects	3	3.24	0.022
Interaction	3	20.40	0.000
Error	831		

## Discussion

Our results show a significant but relatively small variation in male-predominant cuticular hydrocarbons present in a natural population. However, this variation is not associated with mating preferences such as those observed in previous studies with laboratory stocks, in which Canton-S extract or synthetic (*Z*)-7-C_23:1_ was added to males [2], or tester males were perfumed with (*Z*)-7-C_23:1_ rubbed off from other males [18]. The MIs of Canton-S females tested with SC line males varied significantly, but did not indicate a consistent preference for males with a more strongly Canton-S like profile. Moreover, there is no indication of a consistent Canton-S female preference for higher absolute (*Z*)-7-C_23:1_ on males, as has been shown in prior experiments [2], [18]. In fact, SC9 had the lowest amount of (*Z*)-7-C_23:1_ and a low MI, while SC3 had the most (*Z*)-7-C_23:1_ and a relatively high MI. There were also no consistent effects of quantitative variation of (*Z*)-7-C_25:1_ on mating success - SC5 males were the best maters and had the least (*Z*)-7-C_25:1_, but SC9 were the second best maters (and statistically indistinguishable from SC5) and had the most (*Z*)-7-C_25:1_ – more than twice as much as SC5. Overall, we observed no indication of a consistent effect from variation in the male-predominant hydrocarbon mixes of SC line males on their mating success with Canton-S females. Most SC line females mated significantly faster and more frequently with Canton-S than with Tai-Y males. Faster mating with Canton-S is consistent with a preference based on hydrocarbon profiles because the SC line females are all derived from a population with a hydrocarbon mix much more like Canton-S than Tai-Y. However, there were important differences among the SC lines, as indicated by the significant male X female interaction in Table 6. Specifically, SC7 and SC9 females showed a preference for Canton-S males, much like the preference shown by Canton-S females [2], but the hydrocarbon profiles of males from those lines were least like Canton-S. Conversely, females from SC3 and SC5, from lines with hydrocarbon mixes most like Canton-S, showed no preference for males of either strain. Overall, our results suggest that cuticular hydrocarbons provide a general signal to prospective mates, rather than a specific quantitative one in *D. melanogaster*. There does not appear to be substantial precision in females' use of cuticular hydrocarbons to assess potential mates.

Recent studies have shown that cVA is present on the anal-genital region of the male, especially the penis [25], [30], and acts as a pheromonal signal that increases female receptivity [29]. However, we did not observe any positive association between superficial cVA from SC line males and mating effectiveness. Indeed, the highest amount of cVA was washed from males that had the lowest mating effectiveness. Additionally, there is no difference in the amount of cVA washed from Tai-Y and Canton-S males, even though they vary significantly in their mating effectiveness with Canton-S females [2] and with some of the wild-caught females used in this study. Our samples would have included any cVA near the surface and potentially available as a pheromone during courtship, but a hexane wash is likely to have included at least some cVA leached from the interior of the fly and probably not available as a pheromone.

It is not clear how much cVA is actually present on the male's cuticle. Studies that have unambigously detected it on the anal-genital region of the cuticle [25], [30] are not quantitative. Only about 5 ng of cVA remains with the carcass of males after removal of the reproductive tract [28] and Everaerts et al. did not detect any cVA on the surface of the cuticle of naïve males [26]. However, the methods used in the latter two studies might not have detected cVA on the penis, where it seems to be the most plentiful [25] Even if very little is present on the cuticle, cVA could act as a pheromone if it were released during courtship, but the evidence for release of cVA seems equivocal. Some cVA can be detected on the cuticle of males that have just begun mating [31]. This could be cVA that was released during courtship, because transfer of cVA to females does not begin until about a minute or more after mating begins [28]. Presumably cVA present on the male when mating is initiated was there during courtship. However, despite frequent contact between males and females during courtship [12], [13] none is transferred to virgin females [27], [28]. Also, once mating begins, no significant amount of cVA is consistently transferred by contact to females before it is transferred via the reproductive tract [28], [31]. By contrast, transfer of (*Z*)-7-C_23:1_ to females during courtship and mating can be readily detected [26], [31], [33]. Ejima et al. [27] recovered substantial amounts of cVA from filter paper discs on which males had been placed. This could have been cVA available for release during courtship, but not necessarily because males deposit cVA from their reproductive tracts onto the substrate during nonmating activity [22]. It appears that if cVA is present on the male cuticle or released to act as a female-stimulatory pheromone during courtship and mating, it is likely present in relatively low quantities, much lower than those shown to have antiaphrodisiac properties by Zawistowski and Richmond [24].

Our results indicate that the variation in mating success of SC line males with Canton-S females cannot be accounted for by variation in male-predominant cuticular hydrocarbons or cVA. *D. serrata* males facultatively release cuticular hydrocarbons after short (15 minute) visual exposure to females [35]. If such short term changes occurred in *D. melanogaster*, and varied among wild-caught males, it could affect mating success over the course of a 30 minute trial. A comparison of pheromones from naïve and just-mated laboratory males provides a test of this possibility. Just-mated males would have released available pheromones during courtship that led up to the mating, which would be detected in a hexane wash. However, both 30 second and 1 minute hexane washes remove less (*Z*)-7-C_23:1_ from Canton-S males that have just mated than from naïve males [30]. Transfer of some (*Z*)-7-C_23:1_ to females could account for the decrease during mating, but these data do not indicate any increase of (*Z*)-7-C_23:1_ on the cuticle during courtship. A significant increase in (*Z*)-7-C_25:1_ on the cuticle of just-mated males has been reported [31]. However an analysis of (*Z*)-7-C_25:1_ from the chromatograms used by Scott [33] indicates a different trend - a 30 second hexane wash removed 87±5 ng, *N* = 6 of (*Z*)-7-C_25:1_ from naïve males, comparable to the amount removed by a 1 minute wash (Table 2). In comparison, 30 second and 1 minute washes of just-mated males removed 63±15 and 68±13 ng of (*Z*)-7-C_25:1_ respectively (*N* = 6 each), less than would be expected if the pheromone were released during courtship and mating. On this basis, it does not seem that *D. melanogaster* males facultatively release cuticular hydrocarbon pheromones during courtship.

Generally, SC line females mated faster with males from their own strain than with males from either laboratory strain. This suggests that variation in courtship elements other than cuticular hydrocarbons or cVA played a role in mating propensity. Yew et al. [25] recently demonstrated the existence of a specific pheromone present on males and not detected by conventional GC-MS methods. This compound, dubbed CH503 ( =  3-*O*-acetyl-1,3-dihydroxy-octacosa-11,19-diene) is similar to cVA in that it is localized in the ejaculatory bulb, is transferred to females during mating and inhibits male courtship. It differs in that it persists on the female for 10 days after mating [25]. If the pheromonal properties of CH503 included enhancement of female receptivity, as is the case for cVA and (*Z*)-7-C_23:1_, and if it varied among males, it could contribute the variation in mating success.

Courtship elements other than pheromones, such as wing vibration to produce courtship song, licking and abdominal tapping, are important for mating success [13]. We did not quantify the total courtship during the mating tests, but pairs were observed continuously and males generally courted actively, although not constantly, throughout the mating test. It is unlikely that the variation in mating success we observed was due to variation in courtship intensity. First, males from all strains generally courted actively during most of the trial. Second, courtship intensity is not necessarily correlated with mating success. For example Tai-Y males court Canton-S virgin females as actively as Canton-S males do, but are not as successful at mating [12]. Indeed, beyond a certain minimum, courtship intensity may be of limited importance - when quantitative variation in female stimulatory pheromones is used to manipulate courtship intensity, there is no correlation with mating success [17].

Even though variation in courtship song plays a limited role, if any, in the mating asymmetry between Tai-Y and Canton-S [2], it could nonetheless affect mating success among wild-caught lines. Elements of courtship song, for example interpulse interval, can evolve quickly and play a role in female choice in a variety of *Drosophila* species [36], [37], [38], [39]. If present, courtship song variation could have contributed to variation in mating success, especially the strong tendency for SC line females to mate faster with males from their own line than with laboratory males. It could also have contributed to variation in the receptivity of SC line females to Canton-S males, if the similarity of SC line courtship songs to Canton-S was variable.

We observed a strong inverse relationship between the MIs and mating speeds of SC line males with Canton-S females and those of Canton-S males with SC line females. Mating success in *D. melanogaster* depends on both male courtship and female receptivity, with females ultimately determining whether and when mating will occur (see [13], [14], [40]). Populations with less receptive (more discriminating) females would be expected to have males that are more effective courters. Conversely, more receptive females should be accompanied by less effective courters. Our results support this association. Males that have high MIs with Canton-S females are unlikely to court well. The males with the highest MIs came from lines with females that had the lowest MIs when tested with Canton-S males. These are the females that would presumably be the most receptive to mating. Conversely, SC line males with the lowest MIs came from lines with females that had the highest MIs tested with Canton-S males and were presumably the most discriminating.

The observed association between female receptivity and male courtship effectiveness could have been present in the isofemale lines at the time they were collected, which would suggest a direct genetic link between female receptivity and male courtship effectiveness. Alternately, an association could have evolved as the lines were maintained in the laboratory over several generations. Rapid coevolution of male and female elements of mate recognition systems has been demonstrated in laboratory experiments [41]. In either case, it appears that a strong association between female receptivity and male courtship ability either exists in a natural population or is capable of evolving fairly rapidly in isolated populations.
